# Serum microRNA signatures and metabolomics have high diagnostic value in gastric cancer

**DOI:** 10.1186/s12885-018-4343-4

**Published:** 2018-04-13

**Authors:** Hai-Ning Liu, Hao Wu, Yu-Jen Tseng, Yan-Jie Chen, Dan-Ying Zhang, Lin Zhu, Ling Dong, Xi-Zhong Shen, Tao-Tao Liu

**Affiliations:** 1 Department of Gastroenterology, Zhongshan Hospital of Fudan University, Room 207, Building 3, Zhongshan Hospital, Fenglin Road 180#, Xuhui District, Shanghai, China; 2 Department of Geriatrics, Zhongshan Hospital of Fudan University, 180 Fenglin Road, Shanghai, 200032 China; 3 Shanghai Institute of Liver Diseases, Zhongshan Hospital of Fudan University, 180 Fenglin Road, Shanghai, 200032 China

**Keywords:** Stomach neoplasms, Diagnostic test, Meta-analysis, microRNA, Metabolomics, Gas chromatography/mass spectrometry

## Abstract

**Background:**

Many novel diagnostic biomarkers have been developed for gastric cancer (GC) recently. We chose two methods with high diagnostic value, the detection of serum microRNAs and metabolomics based on gas chromatography/mass spectrometry (GC/MS), and aimed to establish appropriate models.

**Methods:**

We reviewed the diagnostic accuracies of all microRNAs identified by previous diagnostic tests. Then appropriate microRNAs and their combinations were validated the diagnostic value. We included 80 patients with GC and 82 healthy controls (HCs) and detected the expression of the microRNAs. GC/MS analysis was conducted, and we used three multivariate statistical analyses to establish diagnostic models. The concentrations of carcinoembryonic antigen (CEA) and carbohydrate antigen 19–9 (CA19–9) were detected for comparison with the novel models.

**Results:**

Sixty-seven published studies and 70 microRNAs were finally included in the systematic review. MiR-18a, miR-19a, miR-21, miR-92a, miR-199a and miR-421 were chosen to further validate their diagnostic efficiencies. Five of those microRNAs in GC patients had significantly different expression. The combination of miR-19a and miR-92a had the highest area under the curve (AUC) at 0.850 with a sensitivity of 91.3% and a specificity of 61.0%. The GC/MS analysis performed an excellent diagnostic value and the AUC reached 1.0.

**Conclusion:**

There is a good potential for microRNAs and GC/MS analysis as new diagnostic methods in view of their high diagnostic value compared with traditional biomarkers.

**Electronic supplementary material:**

The online version of this article (10.1186/s12885-018-4343-4) contains supplementary material, which is available to authorized users.

## Background

Gastric cancer (GC) has the fifth highest cancer morbidity and the third highest mortality rate in the world [1]. The morbidity in East Asia is much higher than that of Caucasians, and the GC patients in China are more than the sum of all other countries [2]. With the control of *Helicobacter pylori* infection, the changes of lifestyle, and the progress of diagnosis and treatment methods, the mortality rate of gastric cancer has gradually decreased [3, 4]. Currently, the diagnosis of GC relies endoscopic biopsy and enhancement CT according to the National Comprehensive Cancer Network (NCCN) Practice Guidelines of gastric cancer. As a supplement to traditional diagnostic methods, discovering circulating biomarkers with high diagnostic value is essential. Novel diagnostic biomarkers for gastric cancer include, but are not limited to, oncogenes, tumor suppressor genes, microRNAs and long non-coding RNAs, DNA methylation and low-molecular-weight metabolites [5–7]. Considering the diagnostic accuracies, advantages and disadvantages, we chose two methods, the detection of serum microRNAs and metabolomics based on gas chromatography/mass spectrometry (GC/MS), to validate their diagnostic efficiencies and attempt to develop appropriate models.

MicroRNAs are non-protein-coding RNAs with small molecular size that regulate target gene expression by binding to their 3′ untranslated region [8]. Thousands of microRNAs have been discovered over the past decade, and quite a few microRNAs have been determined the potential for the diagnosis of GC. Nevertheless, the diagnostic efficiencies of the reported circulating microRNAs are not consistent among studies. It is thus necessary to summarize the diagnostic value of these microRNAs via a systematic review. We did abovementioned work and aimed to overcome the deficiencies of previous systematic reviews and meta-analyses, such as small including article number, single researched microRNA [9], or lack of the information of each microRNA [10–13]. Then we chose six microRNAs with high Youden indexes or area under the curve (AUC) values of the receiver operating curve (ROC) to validate their diagnostic value and establish a diagnostic panel.

Metabolomics is defined as the quantitative measurement of low-molecular-weight metabolites in an organism at a specified time under specific environmental conditions [14]. GC/MS, which is one of metabolomic techniques, has robust results and is widely used in metabolite identification because of its peak resolution, high sensitivity, and reproducibility [15, 16]. Several studies reported its high diagnostic value for GC, and the AUC value usually reached more than 0.90 [17]. As high-throughput experimental data, the results of GC/MS are always processed by multivariate statistical analysis, including the principal component analysis (PCA), partial least squares-discriminate analysis (PLS-DA), and orthogonal partial least squares-discriminant analysis (OPLS-DA). We further validated the diagnostic value of metabolomics and compared the three most frequently used statistical methods.

## Methods

### Study design

First of all, we reviewed the diagnostic accuracies of microRNAs mentioned in previous studies. We searched several relevant databases, including PubMed, Embase, and the Chinese Biomedical Literature Database (CBM) up to Jul 26, 2017. The search strategy was (“stomach neoplasms”[Mesh] OR “gastric cancer” OR “stomach cancer”) AND (miRNA OR microRNA OR miR) AND (blood OR serum OR plasma OR circulating) AND (diagnosis OR diagnostic OR diagnose). There were no language restrictions in searching process. Lists of references of articles were searched manually for additional publications [18].

Then, we selected the microRNAs with high Youden indexes and high AUC values to establish a diagnostic model according to the results of the systematic review. The serum specimens from 80 patients with GC and 82 healthy controls (HCs) were obtained to detect the microRNA levels using quantitative reverse-transcription polymerase chain reaction (qRT-PCR).

Next, we selected 25 GC patients and 30 HCs from the cohort mentioned above with a completely random method and utilized GC/MS to profile the metabolomic signatures.

**Fig. 1:**
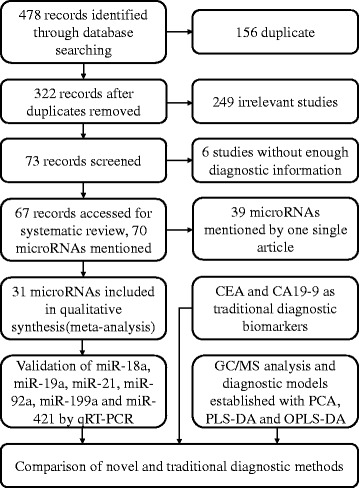
Flow diagram of trial selection. Abbreviations: CEA, carcinoembryonic antigen; CA19–9, carbohydrate antigen 19–9; qRT-PCR, quantitative reverse-transcription polymerase chain reaction; GC/MS, gas chromatography/mass spectrometry; PCA, principal component analysis; PLS-DA, partial least squares-discriminate analysis; OPLS-DA, orthogonal partial least squares-discriminant analysis

Finally, the diagnostic value was compared among the new models and the traditional tumor biomarkers, carcinoembryonic antigen (CEA) and carbohydrate antigen 19–9 (CA19–9). An overview of the study design is illustrated in Fig. 1.

### Inclusion and exclusion criteria of the literature

Studies were included if they met the following inclusion criteria: (1) studies regarding the diagnostic value of microRNAs in GC; (2) blood specimens; and (3) qRT-PCR techniques. Additionally, studies exclusion criteria are: (1) failure to provide enough diagnostic information; (2) duplicate publications reported by identical authorities; and (3) animal or cell studies, letters and reviews.

### Data extraction

Data were extracted independently by two reviewers from all of the included articles: (1) basic characteristics of the studies, including the first author, year of publication, country of publication, ethnicity, sample size, mean or median age, gender, type of specimens (serum or plasma), target microRNAs, and reference control RNA; and (2) diagnostic information of the microRNAs, including the sensitivity, specificity, AUC and expression variation.

### Patients and specimens

We included 80 patients with GC and 82 HCs who were from in Zhongshan Hospital, Fudan University between May 2015 and September 2015. The GC patients were all definitively diagnosed by an endoscopic biopsy. Exclusion criteria were history of other malignant tumors, a surgical operation, radiotherapy or chemotherapy. Healthy individuals were identified by clinical manifestations, histories of diseases and results of blood tests. The samples were centrifuged for 10 min at 820 g and 4 °C to remove residual cell debris, and the supernatants were immediately stored at − 80 °C until further analyses. The serum concentrations of serum CEA and CA19–9 were measured with the electro-chemiluminescence immunoassay.

Approval for the study was given by the Ethics Committee of Zhongshan Hospital of Fudan University, Shanghai. All GC patients and control subjects provided written informed consents before enrollment in this study.

### RNA extraction and reverse transcription

200 μl of the serum samples was spiked with 2 μl of 25 fmol synthetic cel-miR-39 (Tiangen, Beijing, China) as the external reference. Total RNA enriched for small RNAs was isolated simultaneously from the serum with the miRcute microRNA Isolation Kit (Tiangen, Beijing, China) according to the modified manufacturer’s protocol [19]. To determine the purities and concentrations, we utilized a NanoDrop spectrophotometer (NanoDrop, Wilmington, DE, USA) to assess the optical density of the extracted RNA at 260 and 280 nm.

The extracted microRNA was polyadenylated by 20 μl of the poly (A) polymerase. 6 μl of the poly (A) reaction solution was reverse transcribed to cDNA in another 20 μl with miRcute microRNA The First-strand cDNA Synthesis Kit (Tiangen, Beijing, China) following the manufacturer’s instructions. Reverse transcription was run in triplicate.

### Quantitative real-time PCR

The PCR reaction was performed for amplification using the miRcute microRNA qPCR Detection Kit (Tiangen, Beijing, China) on ABI PRISM 7500 Sequence Detection System (Applied Biosystems, Foster City, CA, USA). Each qPCR reaction solution contained diluted cDNA, 2× miRcute microRNA premix (with SYBR and ROX), the manufacturer-provided microRNA-specific forward primer, and a universal reverse primer to a total volume of 20 μl. The qPCR reaction parameters were 94 °C pre-denaturation for 2 min, 45 cycles of 94 °C for 20 s, 60 °C annealing for 34 s, and 72 °C extension for 30 s. A melting curve analysis was accomplished to ensure the specificity of the target PCR product in the end.

The relative expression of the microRNAs was calculated using the equation log_10_ (2^−ΔCT^). The ΔCT was equal to CT values of the microRNAs of interest minus the CT values of the cel-miR-39 [19].

### Specimen processing for metabolomics

For the GC/MS analysis, the serum samples were transferred into glass centrifuge tubes in a 200-μl volume. Each sample was spiked with 200 μl of 2-chloro-phenylalanine (0.3 g/L) as an internal standard and 600 μl of methanol. The mixture was vortexed for 30 s, incubated for 10 min at − 20 °C and then centrifuged for 15 min at 12000×g and 4 °C. Supernatant in an 800-μl volume was collected separately into an ampoule bottle and then evaporated to dryness under a stream of nitrogen gas at 50 °C for around 30 min. Subsequently, 200 μl of a methoxyamine pyridine solution (15 g/L) was put into the ampoule bottle. The mixture was vortexed for 2 min and incubated for 60 min at 37 °C. Next, we added 200 μl of bis-(trimethylsilyl)-trifluoroacetamide (BSTFA) plus 1% trimethylchlorosilane (TMCS), and the mixture was vortexed for 2 min and incubated for 30 min at 100 °C. The methanol, 2-chloro-phenylalanine, methoxyamine and pyridine were bought from Aladdin (Shanghai, China). The BSTFA with 1% TMCS was bought from Sigma-Aldrich (St. Louis, MO, USA). All reaction samples were performed in duplicate.

### GC/MS analysis

The GC/MS analysis was carried out on an Agilent 6980 GC system equipped with a fused-silica capillary column with a 0.25-μm HP-5MS stationary phase (Agilent, Shanghai, China). We used the same operational methods as our previous studies [20].

### Statistical analyses

The statistical analyses were conducted with Stata 12.0 (StataCorp LP, College Station, TX, USA), SIMCA-P 13.0 (Umetrics AB, Umea, Vasterbotten, Sweden) and R software 3.3.3 (R Foundation for Statistical Computing, Vienna, Austria). A *P* value less than 0.05 was considered statistically significant.

Meta-analysis methods for diagnostic tests were used to assess the value of the individual microRNAs to diagnose GC using the sensitivity, specificity and AUC of the summary receiver operator characteristic (SROC). Deeks’ funnel plot was adopted to evaluate the publication bias.

A power analysis was used to obtain the sample size of the GC cases and controls in the microRNA validation phase. Wilcoxon-Mann-Whitney test and Student’s *t*-test were used for the comparison between the patients and the HCs, including the expression of the microRNAs and the concentrations of CEA and CA19–9. The diagnostic efficiencies of the microRNAs were assessed with the sensitivity, specificity and the AUC of the ROC. A logistic regression was utilized to build an appropriate diagnostic model.

The metabolomic information was normalized with “XCMS” package in R software and the data were edited into a two-dimensional matrix, including the mass-to-charge ratio (MZ), retention time (RT) and peak intensity. SIMCA-P software was used to perform multivariate data analyses, including PCA, PLS-DA, and OPLS-DA. A logistic regression was used to investigate the better diagnostic model by combinations of the various components when more than one component was extracted. The metabolites were identified based on the National Institute of Standards and Technology (NIST) mass spectra library through RT and MZ [20]. We screened the significantly different metabolites via the variable importance in the projection (VIP) value (> 1) of the OPLS-DA model and the *P* value (< 0.001) of fold change of Student’s *t*-test between the patients and the HCs.

## Results

### Study selection and literature characteristics

The initial search returned a total of 478 records, among which, 146 were from PubMed, 249 were from Embase, and 83 were from CBM. We removed 156 duplicates, 249 irrelevant studies and six articles that failed to provide enough diagnostic information. Sixty-seven candidate articles were finally enrolled into this systematic review with a total of 5261 GC patients and 4386 healthy controls (Additional file 1: Table S1 and Additional file 2: Table S2).

### Diagnostic value of microRNAs in the literature

There were 70 microRNAs mentioned in the included articles, of which, 39 were studied in one single article. We performed the meta-analyses to represent the diagnostic value of the other 31 microRNAs. The details regarding each microRNA are displayed in Table 1.

**Table 1:** Characteristics of the microRNAs mentioned in the literature

MicroRNA	Expression	GC sample size	Control sample size	Sensitivity (%)	Specificity (%)	AUC	Number of included articles
miR-451	U	144	172	91.2	96.5	0.980	2
miR-183	U	76	26	93.4	92.3	0.978	1
miR-300	U	25	15	96.0	86.7	NA	1
miR-940	D	110	100	73.2	97.9	0.970	1
miR-486	U	144	172	78.1	94.0	0.950	2
miR-421	U	130	107	92.3	79.4	0.940	2
miR-100	U	90	87	86.9	87.3	0.930	2
miR-19a	U	215	241	83.1	89.5	0.930	3
miR-19b	U	215	241	76.6	88.4	0.910	3
miR-627	U	108	96	80.0	89.6	0.900	1
miR-21	U & D	334	250	77.8	87.3	0.900	6
miR-204	D	94	100	73.4	82.6	0.896	1
miR-206	D	150	150	78.0	86.0	0.890	1
miR-200c	U & D	233	145	81.8	81.8	0.890	4
miR-503	D	68	32	96.8	79.4	0.889	1
miR-26a	D	280	280	83.6	81.5	0.882	1
miR-17-3p	U	154	180	69.7	88.9	0.880	2
miR-652	U	108	96	65.7	88.8	0.880	1
miR-130a	U	41	41	77.9	90.2	0.870	1
miR-629	U	108	96	85.2	82.0	0.870	1
miR-196a	U	98	126	69.5	97.6	0.864	1
miR-378	U	40	41	87.5	70.7	0.861	1
miR-101	U	58	60	71.0	88.1	0.857	1
miR-233	U	50	47	81.0	78.0	0.850	1
miR-106b	U	314	275	72.0	84.3	0.850	4
miR-142-3p	D	280	280	74.4	84.1	0.839	1
miR-192	U	29	10	75.9	90.0	0.833	1
miR-18a	U	500	439	73.2	80.8	0.830	6
miR-199a	U	415	296	78.1	74.3	0.830	4
miR-6503-5p	U	361	103	57.9	88.3	0.830	2
miR-200b	D	84	30	62.7	93.4	0.826	1
miR-92a	U	382	665	75.0	77.6	0.820	5
miR-20a	U	615	557	71.8	77.8	0.820	6
miR-223	U	211	211	70.8	78.7	0.820	4
miR-196b	U	98	126	62.2	96.1	0.811	1
miR-16	U	138	189	76.6	87.6	0.810	2
miR-25	U	634	431	67.6	83.5	0.810	5
miR-1	U	240	166	79.2	73.2	0.800	2
miR-375	D	91	91	75.3	73.6	0.800	3
miR-140-5p	U	108	96	81.7	70.0	0.790	1
miR-106a	U & D	287	159	70.0	79.7	0.790	4
miR-744	U	287	228	75.7	72.0	0.790	2
miR-210	U	201	191	76.8	63.9	0.780	2
miR-181b	U	46	21	77.2	72.7	0.770	1
miR-27a	U	149	124	71.8	70.8	0.770	3
miR-17-5p	U & D	313	237	55.3	80.4	0.770	4
miR-195	D	280	280	69.2	75.4	0.765	1
miR-222	U	182	124	62.3	82.6	0.760	2
miR-148a	D	331	331	60.2	76.2	0.760	2
miR-93	U	65	65	81.5	73.8	0.756	1
miR-218	D	60	60	94.3	44.3	0.743	1
miR-23b	U & D	184	104	71.3	88.9	0.730	2
miR-191	U	125	126	61.8	97.6	0.730	2
miR-215	U	29	10	62.1	88.3	0.724	1
miR-371-5p	U	40	41	75.0	63.4	0.715	1
miR-376c	U	68	68	74.0	62.7	0.710	1
miR-187*	U	40	41	82.5	61.0	0.704	1
let-7e	U	68	68	62.1	79.2	0.700	1
miR-320a	D	35	38	65.2	68.2	0.699	1
miR-92b	U	101	91	46.9	88.2	0.690	1
miR-221	U	248	248	63.1	92.1	0.670	3
miR-27b	U	68	68	48.7	82.0	0.660	1
miR-107	U & N	86	86	40.7	80.2	0.660	2
miR-185	U	101	91	46.7	84.9	0.650	1
miR-34a	U	180	106	59.4	67.8	0.645	1
miR-151-5p	U	180	80	61.0	57.0	0.625	1
miR-423-5p	U	180	106	87.3	31.6	0.590	1
miR-103	N	50	50	93.6	24.4	0.548	1
miR-425	U	57	58	31.7	84.2	0.548	1
miR-194	N	50	50	94.2	24.4	0.512	1

We use U to represent the upregulated expression, use D to represent the downregulated expression and use N to represent no significant difference in the GC patients versus the control group. The data on the sensitivity, specificity and AUC were obtained via the meta-analysis when the number of included articles was more than one

*Abbreviations*: *GC* gastric cancer, *AUC* area under the curve, *NA* not available

### Publication bias

Publication bias was assessed with a Deeks’ funnel plot (Additional file 3: Figure S1), and the *P* value of Deeks’ test was 0.24. Therefore, there was no evidence showing that publication bias existed.

### Study population

The clinical and pathological features of the patients and HCs are presented in Table 2. Age was found significant differences between the GC patients and the HCs. We thus performed a covariance analysis. The results suggested that there were no correlations between age and either the expression of the microRNAs, the scores of the components of the metabolomics or the concentrations of CEA and CA19–9.

**Table 2:** Clinical and pathological characteristics of the study population

Variable	Patients (*n* = 80)	Control subjects (*n* = 82)	*P* value
Age (year)	65.1 ± 10.5	34.8 ± 7.3	< 0.001
Gender			0.090
Male	57	48	
Female	23	34	
Tumor size (cm)	4.27 ± 2.62		
> 5	49		
≥ 5	31		
TNM stage			
I	20		
II	18		
III	31		
IV	11		
Histological grade			
I	4		
II	14		
II~III	25		
IV	37		
Lauren classification			
Diffuse	25		
Intestinal	31		
Mixed	24		
Tumor localization			
Cardia	19		
Corpus	8		
Antrum	43		
Whole	10		

*Abbreviation*: *TNM* tumor-node-metastasis

### Expression of microRNAs

**Fig. 2:**
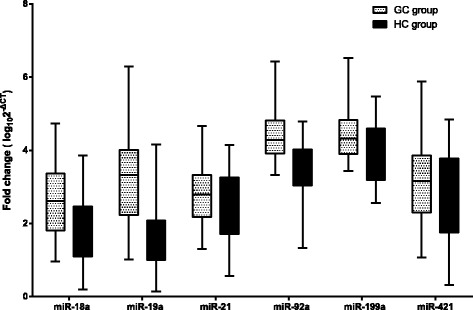
Box plots for the expression of the seven microRNAs. The *P* values of miR-18a, miR-19a, miR-21, miR-92a, miR-199a and miR-421 were <  0.001, < 0.001, 0.024, < 0.001, < 0.001 and 0.098, respectively. The lines within the boxes represent the median values, and the edges of the boxes demonstrate the interquartile ranges. The lines outside the boxes demonstrate the 95% ranges. Abbreviations: GC, gastric cancer; HC, healthy control

MiR-18a, miR-19a, miR-21, miR-92a, miR-199a and miR-421 were chosen in view of their high diagnostic efficiencies in previous studies. The results of the qRT-PCR showed that the serum levels of the microRNAs except miR-421 in the GC patients were significantly higher than those in the HCs (Additional file 4: Table S3 and Fig. 2). The expression of miR-421 wasn’t observed significant difference between the patients and HCs.

### Diagnostic models established using microRNAs

We calculated the sensitivity, specificity, AUC value of each microRNA and their combinations at the optimal cut-off value to find the appropriate diagnostic model (Table 3). The combination of miR-19a and miR-92a had the highest AUC value at 0.850, with a sensitivity of 91.3% and a specificity of 61.0%. The cut-off value of the model was 6.90, according to the formula miR-19a × 0.750 + miR-92a × 1.455.

**Table 3:** Diagnostic value of five single microRNAs and their combinations

MicroRNA(s)	Sensitivity (%)	Specificity (%)	AUC (95% CI)	Cut-off value	Youden index
miR-18a	65.0	69.5	0.731 (0.655, 0.807)	2.201	0.345
miR-19a	80.0	75.6	0.821 (0.757, 0.885)	2.072	0.556
miR-21	77.5	42.7	0.590 (0.502, 0.678)	2.133	0.202
miR-92a	95.0	56.1	0.817 (0.753, 0.881)	3.533	0.511
miR-199a	97.5	36.6	0.684 (0.602, 0.766)	3.530	0.341
miR-421	93.8	20.7	0.556 (0.467, 0.644)	1.525	0.145
**miR-18a + miR-19a**	76.3	80.5	0.833 (0.772, 0.894)	3.600	0.567
miR-18a + miR-21	55.0	80.5	0.729 (0.652, 0.806)	2.454	0.355
miR-18a + miR-92a	96.3	58.5	0.823 (0.760, 0.885)	6.901	0.548
**miR-18a + miR-199a**	78.8	58.5	0.746 (0.672, 0.820)	3.865	0.373
miR-19a + miR-21	78.8	75.6	0.820 (0.756, 0.884)	2.086	0.544
**miR-19a + miR-92a**	91.3	61.0	0.850 (0.794, 0.907)	6.898	0.522
miR-19a + miR-199a	80.0	72.0	0.819 (0.755, 0.883)	3.580	0.520
miR-21 + miR-92a	91.3	63.4	0.820 (0.757, 0.884)	7.300	0.547
miR-21 + miR-199a	93.8	36.6	0.681 (0.600, 0.762)	3.661	0.303
miR-92a + miR-199a	81.3	70.7	0.818 (0.754, 0.881)	7.272	0.520
miR-18a + miR-19a + miR-21	75.0	81.7	0.835 (0.774, 0.896)	3.323	0.567
miR-18a + miR-19a + miR-92a	72.5	85.4	0.857 (0.801, 0.912)	7.780	0.579
miR-18a + miR-19a + miR-199a	78.8	76.8	0.836 (0.775, 0.896)	4.025	0.556
miR-18a + miR-21 + miR-92a	93.8	62.2	0.828 (0.766, 0.890)	6.884	0.559
miR-18a + miR-21 + miR-199a	81.3	57.3	0.746 (0.672, 0.820)	3.845	0.386
miR-18a + miR-92a + miR-199a	98.8	52.4	0.826 (0.764, 0.888)	6.174	0.512
miR-19a + miR-21 + miR-92a	81.3	72.0	0.859 (0.805, 0.913)	7.140	0.532
miR-19a + miR-21 + miR-199a	80.0	72.0	0.820 (0.756, 0.883)	3.410	0.520
miR-19a + miR-92a + miR-199a	72.5	81.7	0.857 (0.802, 0.912)	7.188	0.542
miR-21 + miR-92a + miR-199a	92.5	62.2	0.820 (0.757, 0.884)	6.830	0.547
miR-18a + miR-19a + miR-21 + miR-92a	82.5	73.2	0.861 (0.807, 0.915)	6.938	0.557
miR-18a + miR-19a + miR-21 + miR-199a	75.0	80.5	0.836 (0.775, 0.897)	4.022	0.555
miR-18a + miR-19a + miR-92a + miR-199a	77.5	79.3	0.861 (0.806, 0.915)	6.876	0.568
miR-18a + miR-21 + miR-92a + miR-199a	97.5	58.5	0.830 (0.768, 0.891)	6.166	0.560
miR-19a + miR-21 + miR-92a + miR-199a	80.0	75.6	0.862 (0.808, 0.915)	6.491	0.556
miR-18a + miR-19a + miR-21 + miR-92a + miR-199a	87.5	69.5	0.867 (0.814, 0.920)	5.983	0.570

The bold font indicates that the *P* value of every microRNA in the combination was less than 0.05 in the logistic regression

*Abbreviation*: *AUC* area under the curve, *CI* confidence interval

### Discrepant metabolites and total ion chromatogram

A total of 1118 features were extracted in GC/MS analysis. We found 25 significantly different metabolites (Additional file 5: Table S4). The retention time in the total ion chromatograms was stable with no drift in all of the peaks, which implied that the results were credible.

### Diagnostic models established using metabolomics

We extracted eleven principal components in the PCA model, while eigenvalues in seven of the eleven principal components were more than 1.0. We calculated the diagnostic efficiencies when fitting into one to eleven principal components. When enrolled into more than six principal components, the AUC value reached up to 1.0. Five components were extracted in the PLS-DA model, and the AUC values were all higher than those in the PCA model with the same number of components. Just one factor was extracted in the OPLS-DA model, and the AUC value was 1.0.

**Fig. 3:**
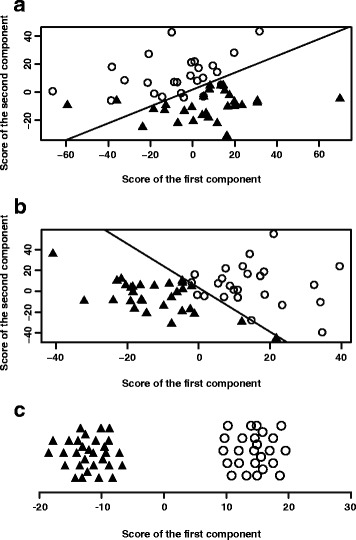
Score plots of the GC/MS analysis in the gastric cancer patients and healthy controls. ○ represents the gastric cancer group. ▲ represents the healthy control group. The scatter plot of the principal component analysis (PCA) (**a**) and partial least squares-discriminate analysis (PLS-DA) (**b**) with two components. The line within the plot represents the optimal cut-off line. **c** The strip chart of the orthogonal partial least squares discriminant analysis (OPLS-DA) with the only component

More details of diagnostic information from the three statistical methods are presented in Table 4 and Fig. 3.

**Table 4:** Diagnostic value of the gas chromatography/mass spectrometry analysis with multivariate statistical analysis methods

Statistical method	Number of components	Sensitivity (%)	Specificity (%)	AUC (95% CI)	Youden index	Cumulative variance
PCA	11	100.0	100.0	1.000 (1.000, 1.000)	1.000	0.445
	10	100.0	100.0	1.000 (1.000, 1.000)	1.000	0.379
	9	100.0	100.0	1.000 (1.000, 1.000)	1.000	0.195
	8	100.0	100.0	1.000 (1.000, 1.000)	1.000	0.153
	7	100.0	100.0	1.000 (1.000, 1.000)	1.000	0.127
	6	92.0	96.7	0.984 (0.960, 1.000)	0.887	0.120
	5	92.0	93.3	0.980 (0.952, 1.000)	0.853	0.086
	4	88.0	96.7	0.976 (0.945, 1.000)	0.847	0.070
	3	92.0	86.7	0.964 (0.924, 1.000)	0.787	0.047
	2	84.0	93.3	0.959 (0.915, 1.000)	0.773	0.039
	1	80.0	63.3	0.707 (0.567, 0.846)	0.433	0.035
PLS	5	100.0	100.0	1.000 (1.000, 1.000)	1.000	0.812
	4	100.0	100.0	1.000 (1.000, 1.000)	1.000	0.791
	3	100.0	100.0	1.000 (1.000, 1.000)	1.000	0.690
	2	92.0	100.0	0.991 (0.974, 1.000)	0.920	0.582
	1	100.0	90.0	0.956 (0.895, 1.000)	0.900	0.501
OPLS	1	100.0	100.0	1.000 (1.000, 1.000)	1.000	0.853

*Abbreviations*: *AUC* area under the curve, *CI* confidence interval, *PCA* principal component analysis, *PLS-DA* partial least squares-discriminate analysis, *OPLS-DA* orthogonal partial least squares-discriminant analysis

### Diagnostic value of traditional tumor biomarkers

The CEA concentration in GC patients was significantly higher than that of HCs (Wilcoxon-Mann-Whitney test, *P* <  0.001). The median concentrations in the patients and HCs were 2.6 (range, 0.5–302.4) and 1.3 (range, 0.3–4.2) μg/L, respectively. For CEA, the sensitivity was 45.0% and the specificity was 95.1% with an AUC of 0.763 (95% CI = 0.686–0.839) when the cut-off value was 2.85 μg/L. When the cut-off value was set at 5 μg/L, which is the traditional upper bound of healthy people, the sensitivity was 22.5%, and the specificity was 100%.

The CA19–9 concentration wasn’t showed significant difference between GC patients and HCs (Wilcoxon-Mann-Whitney test, *P* = 0.203). The median concentrations in the patients and HCs were 9.0 (range, 0.6–423.6) and 7.3 (range, 0.6–26.8) U/ml, respectively. The AUC of CA19–9 was 0.563 (95% CI, 0.468–0.657; sensitivity = 71.3%, specificity  =  44.3%) at the cut-off value of 6.75 U/ml. When the cut-off value was at 37 U/ml, the sensitivity was 12.5%, and the specificity was 100%.

**Fig. 4:**
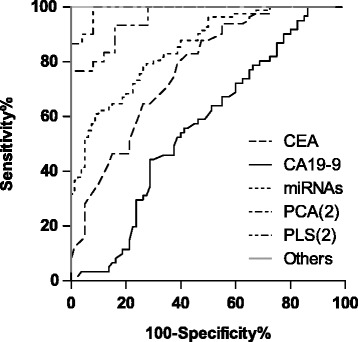
Receiver operating characteristic (ROC) curves. ROC curves of the combination of miR-19a and miR-92a, the PCA and PLS-DA model with two components, CEA, CA19–9 and others for discriminating gastric cancer patients from control subjects. Others include the PCA model with seven principal components, the PLS-DA model with three to five components and the OPLS-DA model with the only component. Abbreviations: PCA, principal component analysis; PLS-DA, partial least squares-discriminate analysis; OPLS-DA, orthogonal partial least squares-discriminant analysis; CEA, carcinoembryonic antigen; CA19–9, carbohydrate antigen 19–9

The ROC curves of the new models and the traditional tumor biomarkers are displayed in Fig. 4.

## Discussion

The development of new technologies has spawned a series of new diagnostic biomarkers. Genomics, microarrays, proteomics, and metabolomics have become general methods for finding novel biomarkers [5]. After reviewing the oncogenes (MMP-9, STC1 and S100A6) [21–23], DNA methylated markers (APBA2, SPG20 and SOX17) [24–26], lncRNAs (UCA1 and LSINCT-5) [27] and the combinations of autoantibody spectrum [28, 29], we found their diagnostic efficiencies not up to expectations. On the contrary, the combinations of microRNAs and metabolomics have the satisfactory diagnostic value constantly [11, 17].

MicroRNA detection has a good many advantages. Compared with long non-coding RNAs and mRNAs, microRNAs are stable and easy to amplify. The stability is reflected at room temperature and even after repeated freeze-thawing [30]. In contrast with gastroscopy, it is inexpensive and non-invasive with almost no complications. Each sample detection for six microRNAs costs approximately 28 dollars in China, which is half of the expense of gastroscopy plus biopsy. The superiority of microRNA detection would be larger in developed countries because of the fancy price of endoscopy. Nevertheless, as nucleic acids, microRNAs cannot be detected directly, and they must first be extracted and reverse transcribed. Furthermore, fold changes and cut-off values are tremendously diverse among different studies because the choice of reference RNA, the dosage of reagents, qPCR detecting instrument and an operating process are not yet standardized. The standardization of protocol is necessary to achieve detection automation and clinical application. The expression of serum microRNAs were altered in various malignant tumors [11, 31–34]. Nevertheless, microRNA diagnostic models may be optimal in determining whether a patient has a malignant tumor. A position diagnosis can be completed through typical clinical manifestations, imaging reports and gastroscopy.

A common research routine of diagnostic test of microRNAs is to screen by the microarray in a small sample size and then validate the results by qRT-PCR in a larger sample size [35]. Other studies validated by qRT-PCR directly after screening from microRNA databases. We chose microRNAs with high diagnostic value via meta-analyses. In view of including more subjects, the selection of microRNAs are more reliable. Three of these microRNAs have potential to become independent biomarkers (AUC > 0.7). It is somewhat disappointing that the combinations of microRNAs didn’t increase the AUC value substantially when we attempted all probable combinations of microRNAs. The combination of miR-18a, miR-19a, miR-21, miR-92a and miR-199a had the AUC value at 0.867 (Table 3). However, it was not significantly different compared to the combination of miR-19a and miR-92a according to the logistic regression.

Similar to previous studies on circulating metabolomics in GC patients, endogenous metabolites, such as amino acids, organic acids, carbohydrates, fatty acids and steroids, were detected with significant differences [36–38]. These varieties suggested metabolism of tumor cells disturbed several metabolic pathways in patients. As a kind of omics technology, metabolomics show a great advantage in diagnosis of GC. It is conceivable that there are hundreds of thousands of low-molecular-weight metabolites that change the concentrations in patients with malignant tumor. Our preliminary experiments even indicated that different malignant tumors could be divided by metabolomics. Besides high diagnostic value, GC/MS analysis also has the affordable price, 72.5 dollars. However, the pretreatment process is not standardized, including the choice of the internal standard and derivatization reagents, the time of each step and the operating order.

Conducting the high-throughput data, the PCA, PLS-DA and OPLD-DA models remain stable when the variables are numerous and the observations are sparse. The results of our study suggest that the OPLS-DA model has the highest AUC and the PCA model ranks the last when including the same number of components. The conclusion could be explained by statistics. PLS-DA and OPLS-DA are supervisory analysis methods, while PCA is non-supervisory. Based on PLS, OPLS further separates the orthogonal variables by an orthogonal signal correction [39, 40]. Although the PCA model is the worst in the three multivariate statistical methods, we could increase the AUC by extracting more principal components. We have noticed that only significantly different metabolites, usually less than ten varieties, were fitted into the diagnostic statistical models in previous studies of metabolomics. We used all 1118 metabolites to construct the model in our study and an internal validation indicated that the models with all metabolites were more robust than those with limited metabolites [41].

Compared with new diagnostic models, CEA showed the inferior diagnostic efficiencies. CEA is better to become a biomarker to predict the recurrence actually [42]. It is interesting that there was no significant differences between GC patients and HCs for CA19–9, which was more commonly used to diagnose pancreatic cancer and colorectal cancer. The cut-off value established by Youden index or Euclidean index of ROC curve could realize more potential to a biomarker than that established by the upper bound of 95% of healthy people.

## Conclusions

In conclusion, the diagnostic value of the new models is higher than that of the traditional biomarkers CEA and CA19–9. We suggest that a GC/MS analysis and a combination of microRNAs allow for the clinical application to diagnosis of GC.

## Additional files

Additional file 1: **Table S1.** List of the included studies. (DOCX 20 kb)

Additional file 2: **Table S2.** Characteristics of the included studies. Abbreviations: GC, gastric cancer; NA, not available. (DOCX 23 kb)

Additional file 3: **Figure S1.** Deeks’ funnel plot for the assessment of publication bias. (DOCX 186 kb)

Additional file 4: **Table S3.** MicroRNA expressions and the results of the statistical test. *Z* value was performed when Wilcoxon-Mann-Whitney test was used in the GC patients versus the control group, and *t* value was performed when Student’s *t*-test was used. Abbreviations: GC, gastric cancer. (DOCX 14 kb)

Additional file 5: **Table S4.** Significantly different metabolites between the gastric cancer patients and the healthy controls. Abbreviations: VIP, variable importance in the projection; MZ, mass-to-charge ratio; RT, retention time. (DOCX 15 kb)

## Abbreviations

APBA2: Amyloid beta precursor protein binding family A member 2
AUC: Area under the curve
BSTFA: Bis-(trimethylsilyl)-trifluoroacetamide
CA19–9: Carbohydrate antigen 19–9
CBM: Chinese Biomedical Literature Database
CEA: Carcinoembryonic antigen
CI: Confidence interval
CT: Cycle threshold or computed tomography
GC: Gastric cancer
GC/MS: Gas chromatography/mass spectrometry
HC: Healthy control
LSINCT-5: Long stress-induced non-coding transcript 5
MMP-9: Matrix metalloproteinase-9
MZ: Mass-to-charge ratio
NCCN: National Comprehensive Cancer Network
NIST: National Institute of Standards and Technology
OPLS-DA: Orthogonal partial least squares-discriminant analysis
PCA: Principal component analysis
PLS-DA: Partial least squares-discriminate analysis
qRT-PCR: Quantitative reverse-transcription polymerase chain reaction
ROC: Receiver operating curve
RT: Retention time
S100A6: S100 calcium binding protein A6
SOX17: SRY-box 10
SPG20: Spastic paraplegia-20
SROC: Summary receiver operator characteristic
STC1: Stanniocalcin-1
TMCS: Trimethylchlorosilane
UCA1: Urothelial cancer associated 1
VIP: Variable importance in the projection
