# Supplementary material for: Serum microRNA signatures and metabolomics have high diagnostic value in gastric cancer
Source: BMC Cancer. 2018 Apr 13;18:415. doi: 10.1186/s12885-018-4343-4 (PMC5899358; doi:10.1186/s12885-018-4343-4)
Supplement: Supplementary file 1 — Table S1. List of the included studies. (DOCX 20 kb) [file 12885_2018_4343_MOESM1_ESM.docx]

**Additional file 1: Table S1 List of the included studies.**

| ID | Article information |
| --- | --- |
| 1 | Fu HL, Yang H, Mao JH, et al. Detection of miRNA-1 in gastric cancer-derived exosome and its clinical significance. Chin J Clin Lab Sci, 2016,34(2):103-106. |
| 2 | Guo F, Yao WY, Dai X, et al. Study on Serum miRNAs as Biomarkers for Early Diagnosis of Gastric Cancer. Chin J Gastroenter, 2014,19(4):198-202. |
| 3 | Wang H, Wang L, Wu Z, et al. Three dysregulated microRNAs in serum as novel biomarkers for gastric cancer screening. Med Oncol, 2014,31(12):298. |
| 4 | Zhu C, Ren C, Han J, et al. A five-microRNA panel in plasma was identified as potential biomarker for early detection of gastric cancer. Br J Cancer, 2014,110(9):2291-2299. |
| 5 | Li H, Wu Q, Li T, et al. The miR-17-92 cluster as a potential biomarker for the early diagnosis of gastric cancer: evidence and literature review. Oncotarget, 2017,8(28):45060-45071. |
| 6 | Zhou H, Guo JM, Lou YR, et al. Detection of circulating tumor cells in peripheral blood from patients with gastric cancer using microRNA as a marker. J Mol Med (Berl), 2010,88(7):709-717. |
| 7 | Tsujiura M, Ichikawa D, Komatsu S, et al. Circulating microRNAs in plasma of patients with gastric cancers. Br J Cancer, 2010,102(7):1174-1179. |
| 8 | Li H, Li T, Liu CH, et al. Circulating miR-17-92 cluster in serum: novel potential biomarkers for gastric cancer. J Mod Oncol, 2014,22(3):581-585. |
| 9 | Shin VY, Ng EK, Chan VW, et al. A three-miRNA signature as promising non-invasive diagnostic marker for gastric cancer. Mol Cancer, 2015,14:202. |
| 10 | Su ZX, Zhao J, Rong ZH, et al. Diagnostic and prognostic value of circulating miR-18a in the plasma of patients with gastric cancer. Tumour Biol, 2014,35(12):12119-12125. |
| 11 | Tsujiura M, Komatsu S, Ichikawa D, et al. Circulating miR-18a in plasma contributes to cancer detection and monitoring in patients with gastric cancer. Gastric Cancer, 2015,18(2):271-279. |
| 12 | Tang GH, Zhang YZ. Expression and significance of miR-18a in serum of elderly patients with gastric cancer. Chinese Journal of Gerontology, 2015(11):2969-2970. |
| 13 | Wang J, Chu YJ, Sun J, et al. Peripheral blood miR-19a and miR-19b in the diagnosis of gastric cancer in the clinical significance. The Journal of Practical Medicine, 2015,31(24):4078-4081. |
| 14 | Zhou X, Zhu W, Li H, et al. Diagnostic value of a plasma microRNA signature in gastric cancer: a microRNA expression analysis. Sci Rep, 2015,5:11251. |
| 15 | Cai H, Yuan Y, Hao YF, et al. Plasma microRNAs serve as novel potential biomarkers for early detection of gastric cancer. Med Oncol, 2013,30(1):452. |
| 16 | Cai H, Yuan Y, Wei X, et al. A study on miR-106b, miR-20a and miR-221 expressions in early gastric cancer. Chin J Gen Surg, 2014,29(2):115-118. |
| 17 | Wu J, Li G, Wang Z, et al. Circulating MicroRNA-21 Is a Potential Diagnostic Biomarker in Gastric Cancer. Dis Markers, 2015,2015:435656. |
| 18 | Li BS, Zhao YL, Guo G, et al. Plasma microRNAs, miR-223, miR-21 and miR-218, as novel potential biomarkers for gastric cancer detection. PLoS One, 2012,7(7):e41629. |
| 19 | Zheng Y, Cui L, Sun W, et al. MicroRNA-21 is a new marker of circulating tumor cells in gastric cancer patients. Cancer Biomark, 2011,10(2):71-77. |
| 20 | Bao XL, Fan H. Value of microRNA-21 in diagnose of early gastric cancer. Chin J Postgrad Med, 2015,38(11):827-829. |
| 21 | Xu W, Fei ZW, Gao M, et al. Diagnostic value of serum miRNA-21 in gastric cancer. J Mod Oncol, 2016,24(22):3592-3595. |
| 22 | Zhuang K, Han K, Tang H, et al. Up-Regulation of Plasma miR-23b is Associated with Poor Prognosis of Gastric Cancer. Med Sci Monit, 2016,22:356-361. |
| 23 | Sun XJ, Zhang Y, Xu YQ. The research on the application value of peripheral blood microRNA-23b as the molecular screening marker of gastric cancer. Chinese Journal of Laboratory Diagnosis, 2014(6):896-898. |
| 24 | Li F, Guo Y, Liu J, et al. The significance of elevated plasma expression of microRNA 106b~25 clusters in gastric cancer. PLoS One, 2017,12(5):e178427. |
| 25 | Liu Y, Zhao GY, Tao LH, et al. Serum microRNA-6503-5p and microRNA-25-3p levels were associated with gastric cancer. Chin J Dig, 2017,37(2):114-117. |
| 26 | Song YQ, Wen WR. Dynamic change and their diagnosis value of serum miR-101 and miR-25 in gastric cancer patients. Journal of Jinan University Natural Science & Medicine Edition, 2016,37(3):259-266. |
| 27 | Qiu X, Zhang J, Shi W, et al. Circulating MicroRNA-26a in Plasma and Its Potential Diagnostic Value in Gastric Cancer. PLoS One, 2016,11(3):e151345. |
| 28 | Park JL, Kim M, Song KS, et al. Cell-Free miR-27a, a Potential Diagnostic and Prognostic Biomarker for Gastric Cancer. Genomics Inform, 2015,13(3):70-75. |
| 29 | Song MY, Pan KF, Su HJ, et al. Identification of serum microRNAs as novel non-invasive biomarkers for early detection of gastric cancer. PLoS One, 2012,7(3):e33608. |
| 30 | Li YQ, Huang PW, Zhu CJ, et al. Diagnostic value of expressions of miR-27a and miR-181b for early detection of gastric cancer. Jiangsu Medical Journal, 2012,38(14):1665-1667. |
| 31 | Niu WW, Yang CC, Duan ZY, et al. The diagnostic value of miRNA-92a combined with micro pepsinogen in gastric carcinoma. Journal of Hebei Medical University, 2017,38(6):638-641, 671. |
| 32 | Zhang X, Niu WW, Yang CC, et al. Clinical significance of abnormal expression of miRNA-92a in serum of gastric cancer patients. Modern Journal of Integrated Traditional Chinese and Western Medicine, 2016,25(18):1953-1956. |
| 33 | Wang LP, Wang QZ, Deng M, et al. Expression and clinical significance of serum microRNA-100 in gastric cancer patients. Chin J Clin Oncol, 2014,41(24):1587-1590. |
| 34 | Oze I, Shimada S, Nagasaki H, et al. Plasma microRNA-103, microRNA-107, and microRNA-194 levels are not biomarkers for human diffuse gastric cancer. J Cancer Res Clin Oncol, 2017,143(3):551-554. |
| 35 | Hou X, Zhang M, Qiao H. Diagnostic significance of miR-106a in gastric cancer. Int J Clin Exp Pathol, 2015,8(10):13096-13101. |
| 36 | Yuan R, Wang G, Xu Z, et al. Up-regulated Circulating miR-106a by DNA Methylation Promised a Potential Diagnostic and Prognostic Marker for Gastric Cancer. Anticancer Agents Med Chem, 2016,16(9):1093-1100. |
| 37 | Ayremlou N, Mozdarani H, Mowla SJ, et al. Increased levels of serum and tissue miR-107 in human gastric cancer: Correlation with tumor hypoxia. Cancer Biomark, 2015,15(6):851-860. |
| 38 | Jiang H, Yu WW, Wang LL, et al. miR-130a acts as a potential diagnostic biomarker and promotes gastric cancer migration, invasion and proliferation by targeting RUNX3. Oncol Rep, 2015,34(3):1153-1161. |
| 39 | Juzenas S, Salteniene V, Kupcinskas J, et al. Analysis of Deregulated microRNAs and Their Target Genes in Gastric Cancer. PLoS One, 2015,10(7):e132327. |
| 40 | Li C, Li JF, Cai Q, et al. miRNA-199a-3p in plasma as a potential diagnostic biomarker for gastric cancer. Ann Surg Oncol, 2013,20 Suppl 3:S397-S405. |
| 41 | Sun Y, Ma J, Hu MJ, et al. Level of miR-183 in peripheral blood of the patients with gastric cancer. J Shanghai Jiaotong Univ (Med Sci), 2017,37(1):75-79. |
| 42 | Liu H, Zhu L, Liu B, et al. Genome-wide microRNA profiles identify miR-378 as a serum biomarker for early detection of gastric cancer. Cancer Lett, 2012,316(2):196-203. |
| 43 | Peng WZ, Ma R, Wang F, et al. Role of miR-191/425 cluster in tumorigenesis and diagnosis of gastric cancer. Int J Mol Sci, 2014,15(3):4031-4048. |
| 44 | Li LP, Long SZ, Gao CP. Expression and clinical significance of microRNA-192 and microRNA-215 in serum of patients with gastric cancer. Chin J Clinicians (Electronic Edition), 2013(12):5223-5227. |
| 45 | Tsai MM, Wang CS, Tsai CY, et al. Circulating microRNA-196a/b are novel biomarkers associated with metastatic gastric cancer. Eur J Cancer, 2016,64:137-148. |
| 46 | Li C, Li JF, Cai Q, et al. MiRNA-199a-3p: A potential circulating diagnostic biomarker for early gastric cancer. J Surg Oncol, 2013,108(2):89-92. |
| 47 | Tang JL, Yan F, Wang XM, et al. Expression of plasma miR-199a-5p and miR-200c-3p and its clinical relevance in gastric carcinoma. Chin J Lab Med, 2015,39(6):402-406. |
| 48 | Chen ZK, Fu SW, Bao ZJ, et al. Changes of serum levels of miR-200b and miR-200c in patients with gastric cancer and its clinical significance. Chin J Biochem Pharm, 2016,36(5):201-203, 206. |
| 49 | Valladares-Ayerbes M, Reboredo M, Medina-Villaamil V, et al. Circulating miR-200c as a diagnostic and prognostic biomarker for gastric cancer. J Transl Med, 2012,10:186. |
| 50 | Lin GY, Zhong HB, Zhang XY, et al. Serum miR-200c in diagnosis of gastric cancer. Zhejiang Medical Journal, 2013,35(10):909-910. |
| 51 | Zhou BZ, Wang DG, Zheng LZ, et al. Changes of serum levels of miR-204 in patients with gastric cancer and its relationship with Helicobacter pylori infection. Chinese Journal of Laboratory Diagnosis, 2017,21(3):485-487. |
| 52 | Hou CG, Luo XY, Li G. Diagnostic and Prognostic Value of Serum MicroRNA-206 in Patients with Gastric Cancer. Cell Physiol Biochem, 2016,39(4):1512-1520. |
| 53 | Qi JP, Shi AP, Feng F, et al. The diagnostic value of serum microRNA-210 in gastric cancer. J Mod Oncol, 2016,24(4):597-599. |
| 54 | Fu Z, Qian F, Yang X, et al. Circulating miR-222 in plasma and its potential diagnostic and prognostic value in gastric cancer. Med Oncol, 2014,31(9):164. |
| 55 | Zhou X, Ji G, Chen H, et al. Clinical role of circulating miR-223 as a novel biomarker in early diagnosis of cancer patients. Int J Clin Exp Med, 2015,8(9):16890-16898. |
| 56 | Liu HN, Zhu XY, Wu H, et al. Diagnostic value and expression of serum miRNA-223 in patients with gastric cancer. Chinese Journal of Clinical Medicine, 2016,23(4):437-439. |
| 57 | Long XE, Dong Z, Yang J, et al. The expression and clinical significance of microRNA-300 in serum of patients with gastric cancer. Modern Practical Medicine, 2014,26(7):796-798, 830. |
| 58 | Xu Q, Dong QG, Sun LP, et al. Expression of serum miR-20a-5p, let-7a, and miR-320a and their correlations with pepsinogen in atrophic gastritis and gastric cancer: a case-control study. BMC Clin Pathol, 2013,13:11. |
| 59 | Zhang WH, Gui JH, Wang CZ, et al. The identification of miR-375 as a potential biomarker in distal gastric adenocarcinoma. Oncol Res, 2012,20(4):139-147. |
| 60 | Zhang WH, Gui JH, Wang CZ, et al. The potential of serum miR-375 as a biomarker of distal gastric adenocarcinoma. Chin J Heal Care Med, 2011,13(5):370-372. |
| 61 | Wu J, Li G, Yao Y, et al. MicroRNA-421 is a new potential diagnosis biomarker with higher sensitivity and specificity than carcinoembryonic antigen and cancer antigen 125 in gastric cancer. Biomarkers, 2015,20(1):58-63. |
| 62 | Zhou H, Xiao B, Zhou F, et al. MiR-421 is a functional marker of circulating tumor cells in gastric cancer patients. Biomarkers, 2012,17(2):104-110. |
| 63 | Konishi H, Ichikawa D, Komatsu S, et al. Detection of gastric cancer-associated microRNAs on microRNA microarray comparing pre- and post-operative plasma. Br J Cancer, 2012,106(4):740-747. |
| 64 | Wu D, Cao G, Huang Z, et al. Decreased miR-503 expression in gastric cancer is inversely correlated with serum carcinoembryonic antigen and acts as a potential prognostic and diagnostic biomarker. Onco Targets Ther, 2017,10:129-135. |
| 65 | Jiang XT, Xia YJ. Significance of serum miRNA-744 detection in the diagnosis of gastric cancer. Zhejiang Practical Medicine, 2016,21(6):394-397. |
| 66 | Liu X, Kwong A, Sihoe A, et al. Plasma miR-940 may serve as a novel biomarker for gastric cancer. Tumour Biol, 2016,37(3):3589-3597. |
| 67 | Zhao GY, Zhang CF, Liu Y, et al. Preliminary study on serum microRNAs as molecular markers for screening gastric cancer. Chin J Dig, 2016,36(8):565-567. |
