# Supplementary material for: Serum microRNA signatures and metabolomics have high diagnostic value in gastric cancer
Source: BMC Cancer. 2018 Apr 13;18:415. doi: 10.1186/s12885-018-4343-4 (PMC5899358; doi:10.1186/s12885-018-4343-4)
Supplement: Supplementary file 2 — Table S2. Characteristics of the included studies. Abbreviations: GC, gastric cancer; NA, not available. (DOCX 23 kb) [file 12885_2018_4343_MOESM2_ESM.docx]

**Additional file 2: Table S2 Characteristics of the included studies.**

| Article Number | First Author | Published Year | Country | Ethnicity | GC group | | | Control group | | | Specimen | MicroRNA | Reference RNA |
| --- | --- | --- | --- | --- | --- | --- | --- | --- | --- | --- | --- | --- | --- |
|  |  |  |  |  | Sample size | Mean age | Gender | Sample size | Mean age | Gender |  |  |  |
| 1 | Fu HL | 2016 | China | Asian | 60 | 64 | 46/14 | 60 | 61 | 37/23 | Serum | miR-1 | miR-16 |
| 2 | Guo F | 2014 | China | Asian | 180 | 59.3 | 120/60 | 106 | 57.2 | 74/32 | Serum | miR-1, miR-20a, miR-34a, miR-423-5p | let-7 |
| 3 | Wang H | 2014 | China | Asian | 50 | NA | 27/23 | 47 | NA | NA | Serum | miR-16, miR-100, miR-233 | U6 |
| 4 | Zhu C | 2014 | China | Asian | 88 | 55.4 | 64/24 | 142 | 53.9 | 101/41 | Plasma | miR-16, miR-25, miR-92a, miR-451, miR-486-5p | cel-miR-39 |
| 5 | Li H | 2017 | China | Asian | 75 | 58.2 | 57/18 | 142 | 47.2 | 87/55 | Serum | miR-17-5p, miR-17-3p, miR-18a-5p, miR-19a-3p, miR-19b-3p, miR-20a-5p, miR-92a-3p | U6 |
| 6 | Zhou H | 2010 | China | Asian | 90 | 61.4 | 63/27 | 27 | NA | NA | Blood | miR-17, miR-106a | U6 |
| 7 | Tsujiura M | 2010 | Japan | Asian | 69 | NA | NA | 30 | NA | NA | Plasma | miR-17-5p, miR-21, miR-106a, miR-106b | U6 |
| 8 | Li H | 2014 | China | Asian | 79 | NA | 56/23 | 38 | NA | NA | Serum | miR-17-5p, miR-17-3p, miR-18a-5p, miR-19a-3p, miR-19b-3p, miR-20a-5p, miR-92a-3p | (Absolute quantification) |
| 9 | Shin VY | 2015 | China | Asian | 108 | 65.2 | 64/44 | 96 | NA | NA | Plasma | miR-18a, miR-140-5p, miR-199a-3p, miR-627, miR-629, miR-652 | U6 |
| 10 | Su ZX | 2014 | China | Asian | 82 | 69 | 48/34 | 65 | 71.2 | 39/26 | Plasma | miR-18a | (Absolute quantification) |
| 11 | Tsujiura M | 2015 | Japan | Asian | 104 | 65.6 | 67/37 | 65 | NA | NA | Plasma | miR-18a | U6 |
| 12 | Tang GH | 2015 | China | Asian | 52 | 68 | 33/19 | 33 | NA | NA | Serum | miR-18a | U6 |
| 13 | Wang J | 2015 | China | Asian | 61 | 57.4 | 40/21 | 61 | 56.1 | 38/23 | Serum | miR-19a, miR-19b | U6 |
| 14 | Zhou X | 2015 | China | Asian | 101 | NA | 62/39 | 91 | NA | 55/36 | Plasma | miR-20a, miR-25, miR-92b, miR-185, miR-210 | cel-miR-39 |
| 15 | Cai H | 2013 | China | Asian | 90 | 46.2 | 66/24 | 90 | 46.1 | 66/24 | Plasma | miR-20a, miR-106b, miR-221 | cel-miR-39 |
| 16 | Cai H | 2014 | China | Asian | 90 | NA | NA | 90 | NA | NA | Plasma | miR-20a, miR-106b, miR-221 | cel-miR-39 |
| 17 | Wu J | 2015 | China | Asian | 50 | NA | 24/26 | 50 | NA | NA | Serum | miR-21 | U6 |
| 18 | Li BS | 2012 | China | Asian | 60 | 54 | NA | 60 | 51 | NA | Plasma | miR-21, miR-218, miR-223 | cel-miR-39 |
| 19 | Zheng Y | 2011 | China | Asian | 53 | NA | 35/18 | 20 | NA | NA | Plasma | miR-21 | U6 |
| 20 | Bao XL | 2015 | China | Asian | 52 | 56.3 | 31/21 | 40 | 55.8 | 23/17 | Plasma | miR-21 | miR-16 |
| 21 | Xu W | 2016 | China | Asian | 50 | NA | 21/29 | 50 | NA | NA | Serum | miR-21 | U6 |
| 22 | Zhuang K | 2016 | China | Asian | 138 | NA | 85/53 | 50 | NA | NA | Plasma | miR-23b | U6 |
| 23 | Sun XJ | 2014 | China | Asian | 46 | 56.3 | 30/16 | 54 | 53.8 | 34/20 | Blood | miR-23b | U6 |
| 24 | Li F | 2017 | China | Asian | 65 | 54.1 | 50/15 | 65 | 56.2 | 50/15 | Plasma | miR-25, miR-93, miR-106b | U6 |
| 25 | Liu Y | 2017 | China | Asian | 322 | 64.5 | 240/82 | 73 | 59.7 | 45/28 | Serum | miR-25-3p, miR-6503-5p | cel-miR-39 |
| 26 | Song YQ | 2016 | China | Asian | 58 | 51.4 | 32/26 | 60 | 50.64 | 35/25 | Serum | miR-25, miR-101 | (Absolute quantification) |
| 27 | Qiu X | 2016 | China | Asian | 280 | 63.3 | 177/103 | 280 | 63.2 | 177/103 | Plasma | miR-26a, miR-142-3p, miR-148a, miR-195 | cel-miR-39 |
| 28 | Park JL | 2015 | Korea | Asian | 35 | 51.8 | 18/17 | 35 | 48.9 | 18/17 | Plasma | miR-27a | U6 |
| 29 | Song MY | 2012 | China | Asian | 68 | 60.4 | NA | 68 | 60.3 | NA | Serum | miR-27a, miR-27b, miR-191, miR-221, miR-222, miR-376c, miR-744, miR-let-7e | cel-miR-39 |
| 30 | Li YQ | 2012 | China | Asian | 46 | 58.8 | 30/16 | 21 | 57 | 14/7 | Plasma | miR-27a, miR-181b | cel-miR-39 |
| 31 | Niu WW | 2017 | China | Asian | 60 | 58.5 | 36/24 | 303 | 56.8 | 147/156 | Plasma | miR-92a | U6 |
| 32 | Zhang X | 2016 | China | Asian | 80 | 59.9 | 45/35 | 40 | 55.5 | 22/18 | Plasma | miR-92a | U6 |
| 33 | Wang LP | 2014 | China | Asian | 40 | 63 | 27/13 | 40 | NA | NA | Serum | miR-100 | (Absolute quantification) |
| 34 | Oze I | 2017 | Japan | Asian | 50 | NA | 29/21 | 50 | NA | 29/21 | Plasma | miR-103, miR-107, miR-194 | cel-miR-39 |
| 35 | Hou X | 2015 | China | Asian | 80 | 68 | 46/34 | 80 | 67 | 44/36 | Plasma | miR-106a | U6 |
| 36 | Yuan R | 2016 | China | Asian | 48 | NA | 38/10 | 22 | NA | NA | Plasma | miR-106a | U6 |
| 37 | Ayremlou N | 2015 | Iran | Caucasian | 36 | 59.3 | 24/12 | 36 | NA | NA | Serum | miR-107 | 5srRNA |
| 38 | Jiang H | 2015 | China | Asian | 41 | 54 | NA | 41 | NA | NA | Serum | miR-130a | U6 |
| 39 | Juzenas S | 2015 | Lithuania | Caucasian | 51 | 68.3 | 34/17 | 51 | 56.5 | 20/31 | Plasma | miR-148a-3p, miR-223-3p, miR-375 | miR-16 |
| 40 | Li C | 2013 | China | Asian | 180 | 58.1 | 124/56 | 80 | 58.9 | NA | Plasma | miR-151-5p, miR-199a-3p | U6 |
| 41 | Sun Y | 2017 | China | Asian | 76 | 62 | 53/26 | 26 | 59 | 16/10 | Serum | miR-183 | U6 |
| 42 | Liu H | 2012 | China | Asian | 40 | 56 | NA | 41 | 58 | NA | Serum | miR-187*, miR-371-5p, miR-378 | U6 |
| 43 | Peng WZ | 2014 | China | Asian | 57 | NA | NA | 58 | NA | NA | Serum | miR-191, miR-425 | miR-16 |
| 44 | Li LP | 2013 | China | Asian | 29 | 61.3 | 23/6 | 10 | NA | NA | Serum | miR-192, miR-215 | U6 |
| 45 | Tsai MM | 2016 | China | Asian | 98 | 64.1 | 57/41 | 126 | 66.4 | 60/66 | Plasma | miR-196a, miR-196b | miR-16 |
| 46 | Li C | 2013 | China | Asian | 80 | 56.7 | 55/35 | 70 | 58.9 | 45/25 | Plasma | miR-199-3p | U6 |
| 47 | Tang JL | 2015 | China | Asian | 47 | 60.5 | 34/13 | 50 | 59.7 | 32/18 | Plasma | miR-199a-5p, miR-200c-3p | U6 |
| 48 | Chen ZK | 2016 | China | Asian | 84 | 59.7 | 49/35 | 30 | 57.4 | 18/12 | Serum | miR-200b, miR-200c | U6 |
| 49 | Valladares-Ayerbes M | 2012 | Spain | Caucasian | 52 | 65.9 | 42/10 | 15 | 65.3 | 12/3 | Blood | miR-200c | U6 |
| 50 | Lin GY | 2013 | China | Asian | 50 | 62.8 | 36/14 | 50 | 63 | 37/13 | Serum | miR-200c | U6 |
| 51 | Zhou BZ | 2017 | China | Asian | 94 | 63.3 | 53/41 | 100 | 58.9 | 55/45 | Serum | miR-204 | U6 |
| 52 | Hou CG | 2016 | China | Asian | 150 | 59.8 | 98/52 | 150 | NA | NA | Serum | miR-206 | cel-miR-39 |
| 53 | Qi JP | 2016 | China | Asian | 100 | NA | NA | 100 | NA | NA | Serum | miR-210 | U6 |
| 54 | Fu Z | 2014 | China | Asian | 114 | NA | 54/60 | 56 | NA | NA | Plasma | miR-222 | U6 |
| 55 | Zhou X | 2015 | China | Asian | 50 | 57.8 | 34/16 | 50 | 56.8 | 32/18 | Plasma | miR-223 | U6 |
| 56 | Liu HN | 2016 | China | Asian | 50 | 64.5 | 33/17 | 50 | 34.8 | 23/27 | Serum | miR-223 | cel-miR-39 |
| 57 | Long XE | 2014 | China | Asian | 25 | 66.3 | 17/8 | 15 | 64.4 | 10/5 | Serum | miR-300 | U6 |
| 58 | Xu Q | 2013 | China | Asian | 35 | NA | 0/35 | 38 | NA | 0/38 | Serum | miR-320a | (Absolute quantification) |
| 59 | Zhang WH | 2012 | China | Asian | 20 | 60.9 | 15/5 | 20 | NA | NA | Serum | miR-375 | U6 |
| 60 | Zhang WH | 2011 | China | Asian | 20 | 45.2 | 13/7 | 20 | 39.7 | 10/10 | Serum | miR-375 | U6 |
| 61 | Wu J | 2015 | China | Asian | 90 | NA | 44/46 | 90 | NA | NA | Serum | miR-421 | U6 |
| 62 | Zhou H | 2012 | China | Asian | 40 | 64.9 | 29/11 | 17 | NA | NA | Blood | miR-421 | U6 |
| 63 | Konishi H | 2012 | Japan | Asian | 56 | 66 | 31/25 | 30 | NA | NA | Plasma | miR-451, miR-486 | U6 |
| 64 | Wu D | 2017 | China | Asian | 68 | NA | 43/25 | 32 | NA | 21/11 | Serum | miR-503 | U6 |
| 65 | Jiang XT | 2016 | China | Asian | 219 | 52.1 | 144/75 | 160 | NA | NA | Serum | miR-744 | U6 |
| 66 | Liu X | 2016 | China | Asian | 110 | 67 | 74/36 | 100 | 56 | 59/41 | Plasma | miR-940 | miR-16 |
| 67 | Zhao GY | 2016 | China | Asian | 39 | 61 | 27/12 | 30 | 60.8 | 20/10 | Serum | miR-6503-5p | cel-miR-39 |

Abbreviations: GC, gastric cancer; NA, not available.
