# Supplementary figures and images for: Serum microRNA signatures and metabolomics have high diagnostic value in gastric cancer
Source: BMC Cancer. 2018 Apr 13;18:415. doi: 10.1186/s12885-018-4343-4 (PMC5899358; doi:10.1186/s12885-018-4343-4)

**Additional file 3: Figure S1 Deeks’ funnel plot for the assessment of publication bias.**


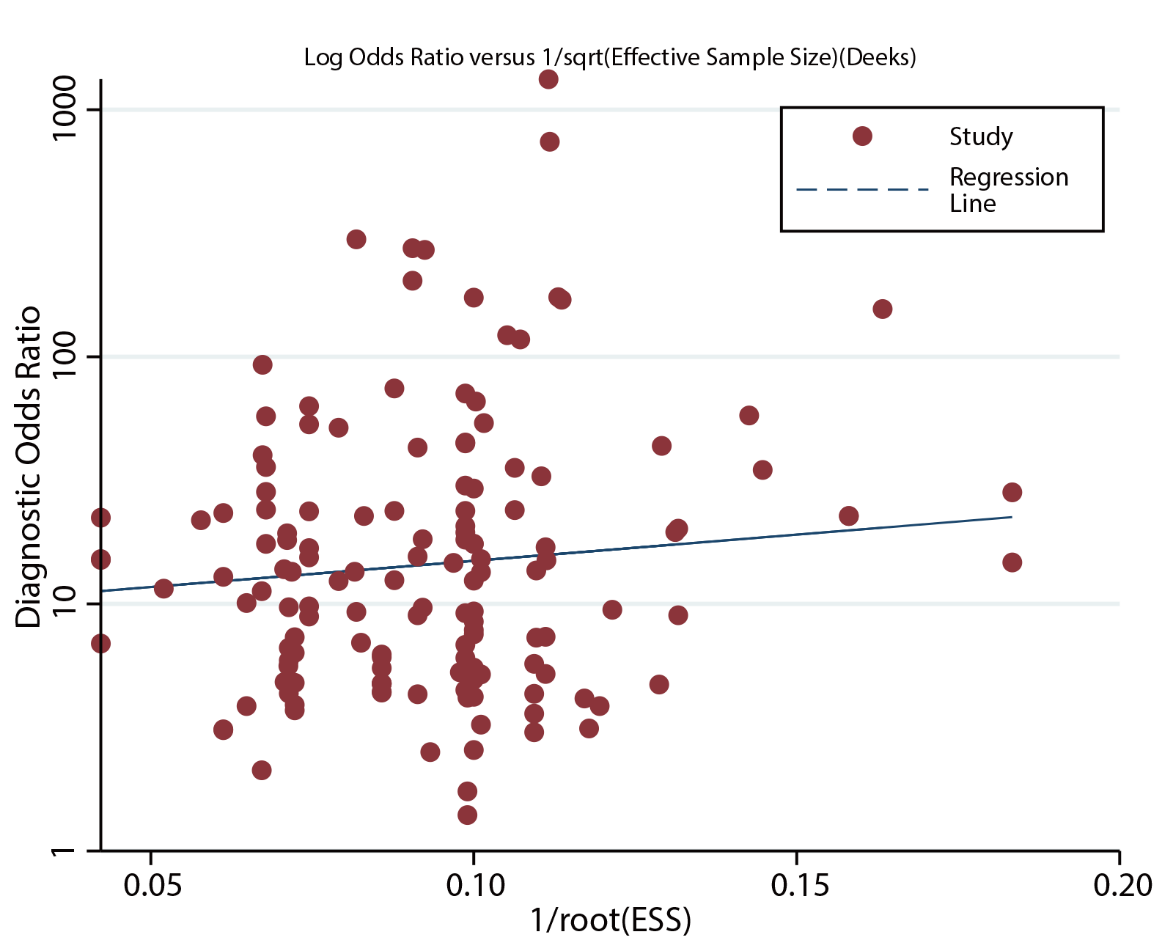

Supplement: Supplementary file 3 — Figure S1. Deeks’ funnel plot for the assessment of publication bias. (DOCX 186 kb) [file 12885_2018_4343_MOESM3_ESM.docx]
