# Supplementary material for: Serum microRNA signatures and metabolomics have high diagnostic value in gastric cancer
Source: BMC Cancer. 2018 Apr 13;18:415. doi: 10.1186/s12885-018-4343-4 (PMC5899358; doi:10.1186/s12885-018-4343-4)
Supplement: Supplementary file 4 — Table S3. MicroRNA expressions and the results of the statistical test. Z value was performed when Wilcoxon-Mann-Whitney test was used in the GC patients versus the control group, and t value was performed when Student’s t-test was used. Abbreviations: GC, gastric cancer. (DOCX 14 kb) [file 12885_2018_4343_MOESM4_ESM.docx]

**Additional file 4: Table S3 MicroRNA expressions and the results of the statistical test.**

|  | Fold Change | | *t* or *Z* value | *P* value |
| --- | --- | --- | --- | --- |
|  | GC group | Control group |  |  |
| miR-18a | 2.66 ± 1.02 | 1.80 ± 0.93 | t = -5.619 | <0.001 |
| miR-19a | 3.23 ± 1.26 | 1.70 ± 1.06 | Z = -7.045 | <0.001 |
| miR-21 | 2.78 ± 0.87 | 2.45 ± 0.96 | t = -2.276 | 0.024 |
| miR-92a | 4.43 ± 0.74 | 3.47 ± 0.78 | Z = -6.971 | <0.001 |
| miR-199a | 4.46 ± 0.85 | 3.88 ± 0.81 | Z = -4.040 | <0.001 |
| miR-421 | 3.11 ± 1.11 | 2.80 ± 1.21 | Z = -1.223 | 0.098 |

*Z* value was performed when Wilcoxon-Mann-Whitney test was used in the GC patients versus the control group, and *t* value was performed when Student’s *t*-test was used.

Abbreviations: GC, gastric cancer.
