# Supplementary material for: Serum microRNA signatures and metabolomics have high diagnostic value in gastric cancer
Source: BMC Cancer. 2018 Apr 13;18:415. doi: 10.1186/s12885-018-4343-4 (PMC5899358; doi:10.1186/s12885-018-4343-4)
Supplement: Supplementary file 5 — Table S4. Significantly different metabolites between the gastric cancer patients and the healthy controls. Abbreviations: VIP, variable importance in the projection; MZ, mass-to-charge ratio; RT, retention time. (DOCX 15 kb) [file 12885_2018_4343_MOESM5_ESM.docx]

**Additional file 5: Table S4 Significantly different metabolites between the gastric cancer patients and the healthy controls.**

| Significantly different metabolites | VIP | MZ | RT | Fold change |
| --- | --- | --- | --- | --- |
| Propanoic acid | 1.283 | 45 | 5.82 | -0.329 |
| 3-Trimethylsiloxymyristic acid | 1.345 | 373.1726 | 11.1 | -0.553 |
| (-)-Cholesterol | 1.474 | 178.1842 | 32.28 | -0.644 |
| Silane | 1.597 | 138.1256 | 32.54 | -0.725 |
| Tocopherol | 1.544 | 223.1481 | 30.32 | -0.716 |
| Cyclopentasiloxane | 1.483 | 72.1 | 7.04 | -0.597 |
| Silanol | 1.58 | 89.8624 | 9.27 | -0.9 |
| l-Threonine | 1.402 | 175.1514 | 9.52 | -1.592 |
| Hexadecanoic acid | 1.352 | 374.4007 | 26.67 | -0.876 |
| Octadecanoic acid | 1.241 | 400.4019 | 28.12 | -0.707 |
| Glucose oxime | 1.77 | 170.1194 | 18.06 | 0.35 |
| Hexadecane | 1.575 | 218.1946 | 26.45 | -0.921 |
| D-Glucose | 1.867 | 432.2993 | 18.11 | 0.578 |
| Heptacosane | 1.284 | 169.1561 | 17.96 | 0.425 |
| Butanoic acid | 1.254 | 392.1724 | 7.42 | 1.69 |
| Phosphoric acid | 1.222 | 242.0962 | 8.11 | -0.58 |
| D-Mannose | 1.884 | 310.2081 | 18.16 | 0.537 |
| D-Mannitol | 1.442 | 481.3809 | 18.38 | 0.486 |
| D-Galactose | 2.078 | 434.2989 | 18.11 | 2.245 |
| Undecane | 1.743 | 56.0973 | 5.72 | -0.378 |
| Hentriacontane | 1.063 | 397.3975 | 27.96 | -0.515 |
| Ethanamine | 1.773 | 58.0991 | 23.69 | -1.276 |
| Tetratriacontane | 1.613 | 410.3859 | 27.92 | -0.751 |
| Pentacosane | 1.731 | 412.3807 | 27.99 | -0.827 |
| Heptasiloxane | 1.877 | 415.3654 | 28.02 | -1.02 |

Abbreviations: VIP, variable importance in the projection; MZ, mass-to-charge ratio; RT, retention time.
